# Interaction between polyphenols intake and PON1 gene variants on markers of cardiovascular disease: a nutrigenetic observational study

**DOI:** 10.1186/s12967-016-0941-6

**Published:** 2016-06-23

**Authors:** Federica Rizzi, Costanza Conti, Elena Dogliotti, Annalisa Terranegra, Erika Salvi, Daniele Braga, Flavia Ricca, Sara Lupoli, Alessandra Mingione, Francesca Pivari, Caterina Brasacchio, Matteo Barcella, Martina Chittani, Francesca D’Avila, Maurizio Turiel, Monica Lazzaroni, Laura Soldati, Daniele Cusi, Cristina Barlassina

**Affiliations:** Genomics and Bionformatics Unit, Department of Health Sciences, Università degli Studi di Milano, viale Ortles 22/4, Milan, Italy; Kos Genetic srl, Milan, Italy; Division of Nephrology, ASST Santi Paolo e Carlo, Via Antonio di Rudinì 8, Milan, Italy; Fondazione Umberto Veronesi, Milan, Italy; Division of Translational Medicine, Sidra Medical Research Center, Doha, Qatar; Sport Medicine Division, Department of Public Health, University Federico II, Naples, Italy; Department of Health Sciences, Università degli Studi di Milano, Via Antonio di Rudinì 8, Milan, Italy; IRCCS Galeazzi Orthopedic Institute, Via Riccardo Galeazzi, 4, Milan, Italy; Laboratory of Clinical Pathology and Medical Genetics, Foundation IRCCS Neurological Institute C. Besta, Milan, Italy; Institute of Biomedical Technologies, Italian National Centre of Research, Segrate, Milan, Italy

**Keywords:** Nutrigenomics, Lipid profile, Anthocyanins, Polyphenols, HDL, Antioxidants, Genetic variants, Gene diet interaction, PON1 gene

## Abstract

**Background:**

Paraoxonase 1 (PON1) gene polymorphisms and polyphenols intake have been reported independently associated to lipid profile and susceptibility to atherosclerosis and cardiovascular disease. However, the interaction between these factors remains to be investigated. We performed an observational nutrigenetic study to examine whether the interaction between polyphenols and anthocyanins intake and PON1 genetic variants can modulate biomarkers of cardiovascular health in an Italian healthy population.

**Methods:**

We recruited 443 healthy volunteers who participated in the EC funded ATHENA project (AnThocyanin and polyphenols bioactive for Health Enhancement through Nutritional Advancement). Data collection included detailed demographic, clinical, dietary, lifestyle, biochemical and genetic data. Polyphenols and anthocyanins intake was measured by 24 h dietary recall repeated three times a year in order to get seasonal variations. We tested the interaction between 18 independent tagging SNPs in PON1 gene and polyphenols intake on HDL, LDL, cholesterol, triglycerides and atherogenic index of plasma.

**Results:**

Without considering the genetic background, we could not observe significant differences in the lipid profile between high and low polyphenols and anthocyanins intake. Using a nutrigenetic approach, we identified protective genotypes in four independent polymorphisms that, at Bonferroni level (p ≤ 0.0028), present a significant association with increased HDL level under high polyphenols and anthocyanins intake, compared to risk genotypes (rs854549, Beta = 4.7 per C allele; rs854552, Beta = 5.6 per C allele; rs854571, Beta = 3.92 per T allele; rs854572, Beta = 3.94 per C allele).

**Conclusions:**

We highlight the protective role of genetic variants in PON1 towards cardiovascular risk under high polyphenols and anthocyanins consumption. PON1 variants could represent novel biomarkers to stratify individuals who might benefit from targeted dietary recommendation for health promotion and strategies of preventive medicine.

**Electronic supplementary material:**

The online version of this article (doi:10.1186/s12967-016-0941-6) contains supplementary material, which is available to authorized users.

## Background

Cardiovascular disease (CVD) is responsible of approximately one-third of deaths in the world and its risk factors continue to increase [1, 2]. Since 2007, the ‘Grand challenges in chronic non-communicable diseases’ outlined that promoting salutary lifestyle and increasing the availability and consumption of healthy food have a key role in the protection against chronic diseases, a global epidemic and economic burden for our society [3, 4].

The physiological response to diet can be modulated by the genetic background, with common DNA variants having the potential to affect specific functional pathways and making subjects more or less responder to a specific diet [5]. For instance, changes in plasma lipid concentration in response to dietary fat intake are largely genetically controlled [6, 7].

Epidemiological studies support the preventive effect of anthocyanins and polyphenols towards the onset of CVD [8–11] through their great antioxidant and antiatherosclerotic activity [12, 13]. Animal and in vitro studies are in favor of their potential to influence lipid profile, a commonly used biomarker of cardiovascular risk [13]. They can slow or inhibit the absorption of lipids and glucose in the intestine and inhibit cholesterol synthesis [14], which results in a decrease in serum triglyceride, total cholesterol and non-HDL cholesterol and in increase in serum HDL. Moreover, they promote reverse cholesterol transport, which also contributes to their effect on lipid profile [12, 15, 16].

Polyphenols are the most abundant antioxidants in the diet. Their dietary intake can be as high as 1 g/dl, much higher than that of all other known dietary antioxidants. Their main dietary sources are fruits and plant-derived beverages (fruit juices, tea, coffee and red wine), vegetables, cereals, chocolate and dry legumes.

Anthocyanins are plant pigments members of the flavonoid family of polyphenols, providing the bright red–orange to blue–violet colors present in many fruits and vegetables.

Interventional studies have consistently reported that polyphenols and athocyanins-rich diets can modulate paraoxonase 1 (PON1) activity and/or the level of PON1 expression [17–19]. PON1 is a member of a gene family, which also comprises PON2 and PON3, all clustered in tandem on the long arm of human chromosome 7 (q21.22). PON1 enzyme is a glycoprotein with hydrolytic activity [20], which associates with HDL in the circulation. It metabolizes a broad variety of substrates, and is a primary determinant of the antioxidant and anti-inflammatory activity of HDL, promoting the HDL-mediated macrophage cholesterol efflux [15, 16]. As such, PON1 plays a relevant role in determining susceptibility to atherosclerosis and cardiovascular disease [17, 21, 22]. It is recognized that PON1 activity is genetically regulated, with Single Nucleotide Polymorphisms (SNPs) exhibiting strong association with its arylesterase and paraoxanase activities [17, 20, 23]. By the way, the wide range of serum PON1 activity among individuals is only partially explained by genetic polymorphisms. The presence of additional factors known to modulate PON1 activity and HDL (e.g. dietary factors, lifestyle, statins, etc.) also needs to be taken into account.

The EC funded the Athena project (FP7-KBBE-2009-3) “Anthocyanin and polyphenols bioactive for health enhancement through nutritional advancement”. Within this project, we conducted an observational study with the aim to explore the relationship between SNPs in PON1 and lipid profile as biomarker of cardiovascular health, taking into consideration polyphenols and anthocyanins consumption. We selected HDL, LDL, total cholesterol, triglycerides and the atherogenic index of plasma (AIP) as common biomarkers, broadly used in clinical practice and with limited inter- and intra assay variability.

## Methods

### Study design and participants

We performed an observational nutrigenetic study on 500 volunteers (age range 20–85 years), as defined in the ATHENA project. Participants were recruited in two healthcare centers in Milano (Italy), ASST Santi Paolo e Carlo, Division of Nephrology, and IRCCS Galeazzi Orthopedic Institute, from June 2012 to December 2013. The subjects participating to the study were required to be in good health, defined according to individual medical history and to routine biochemical, urine and instrumental examination (electrocardiogram, ambulatory blood pressure monitoring, echocardiography, carotid artery and renal ultrasound). Exclusion criteria included evidence of cardiovascular or coronary artery disease, neoplasm, psychosis and diabetes mellitus. The ethic committees of both centres approved the study, (Register 441 signed off on 9 June 2011 and Register 45-1250-ATHENA signed off on 22 February 2013, respectively), in accordance with principles of the Declaration of Helsinki and all participants gave written informed consent.

Volunteers underwent a rigorous clinical examination. At first visit, we collected baseline anthropometric and metabolic measurements and information on diet, personal medical history and lifestyle. A blood sample was also taken for the genetic analysis. We repeated the nutritional interview four times throughout the year in order to get seasonal diet variations. Subjects with less than 3 dietary recall interviews were excluded from the analysis. We then calculated the average annual dietary anthocyanins and polyphenols intake for each participant, assuming that this reflects the individual dietary habit. We divided participants in two groups according to their low or high antioxidant intake, considering anthocyanin and polyphenols separately.

### Nutritional data collection

Experienced dieticians carried out the dietary assessment through 24 h recall interviews. Food portions were defined using a Food Atlas (edited by Scotti-Bassani), including 99 tables with photographs. 24 h recall data were recorded at least three times in a year, once during the first clinical visit and then every 3–4 month.

For data collection and dietary nutrient composition estimate we used the Diet Monitoring Solution (DMS) software, designed and developed by KOS genetic s.r.l., in the context of the Athena project [24]. DMS calculates macro- and micro-nutrients composition using composition tables from the Food Composition Database for Epidemiological Studies in Italy (BDA-IEO), integrated with tables from the USDA Database for the Flavonoid Content and from the Phenol Explorer 2.0 Database.

### Serum lipids analysis

Determination of HDL, LDL, total cholesterol and triglycerides was carried out on automatic clinical chemistry instrumentation (Abbott Architect 8000). Samples were analyzed in batches to limit the analytical variations, after storage at −40 °C for a time not exceeding one month. AIP was calculated as the logarithmically transformed ratio of triglycerides and HDL concentration in plasma [25].

### Genotyping and imputing

Genomic DNA was extracted from peripheral blood using the commercial NucleoSpin Blood Kit by Macherey–Nagel (Macherey–Nagel, Düren, Germany) following the manufacturer’s protocol, and stored at −20°. Forty hundred and seventy-one samples were genotyped using the Illumina HumanCore array (Illumina Inc, San Diego, CA, USA) that contains about 300 K highly informative genome-wide tag-SNPs including indels and updated exome-focused markers. Genotypes were assigned all simultaneously using Genome Studio software (version V2011.1 genotyping module 1.9.4). Genome-wide imputation was performed using MINIMAC software [26, 27] and HapMap CEU haplotypes (release 22) as reference. Measured SNPs with call rate ≥99 % and minor allele frequency (MAF) ≥1 % were included in the dataset. Imputed SNPs with low imputation quality (r2 <0.7) were not used in the association analysis.

PON1 gene maps to chromosome 7 (chr7:94764924-94791780). To select SNPs for the analyses, we first reviewed all SNPs in this gene, including SNPs mapping 1 Kb up-stream and down-stream the 5′ and the 3′ of the gene. We selected 18 independent tagging SNPs in high linkage disequilibrium (r2 ≥0.80) with 18 neighboring SNPs (Table 1). All SNPs complied with Hardy–Weinberg equilibrium. The 18 selected SNPs covered the entire gene and the 3′ and 5′ flanking regions.

**Table 1:** Tagging SNPs analysed in PON1

SNP	Alleles (minor/major)	MAF^a^	Position (bp)	Location^b^	Tagged SNPs^c^
rs854549	A/C	0.39	94,764,521	3downstream	
rs3735590	A/G	0.05	94,765,431	3downstream	rs3917572
rs854551	A/G	0.14	94,765,613	3utr	rs854550
rs854552	C/T	0.20	94,765,860	3utr	
rs3917567	C/T	0.04	94,768,021	Intronic	rs3917556,rs3917527,rs3917551,rs3917548,rs3917541,rs3917569,rs2158155
rs854555	A/C	0.33	94,768,327	Intronic	
rs3917550	A/G	0.10	94,772,509	Intronic	
rs662	C/T	0.30	94,775,382	Coding	rs2057681,rs2269829
rs3917538	A/G	0.24	94,775,829	Intronic	
rs2074354	A/G	0.13	94,778,523	Intronic	rs3917577,rs3917586
rs854560	T/A	0.44	94,784,020	Missense	rs705378,rs854561
rs3917498	T/G	0.35	94,784,191	Intronic	
rs2074351	A/G	0.30	94,785,735	Intronic	
rs2272365	C/A	0.19	94,786,562	Intronic	
rs854569	T/G	0.14	94,787,991	Intronic	
rs3917477	G/A	0.04	94,789,902	Intronic	rs3917476
rs854571	T/C	0.26	94,792,555	5upstream	rs854570
rs854572	C/G	0.39	94,792,632	5upstream	

*Bp* base pair, *MAF* minor allele frequency

^a^Allele frequencies were calculated in the studied population sample

^b^Position and location were taken from the NCBI build 36 (hg18)

^c^Tagged SNPs present a r2 >0.80 with analyzed PON1 markers

### Statistical analysis

The genotyped sample underwent quality control in accordance with the protocol written by Anderson et al. [28]. The degree of recent shared ancestry for a pair of individuals was estimated using genome-wide identity by descent (IBD) as implemented in PLINK software v1.07 [29]. We assessed population stratification with principal component analysis (PCA) as implemented in SNP and Variation Suite v8.x (Golden Helix, Inc., Bozeman http://www.goldenhelix.com) [30]. We selected the first 10 PCs to include as covariates in the linear regression model.

We performed a quantitative trait interaction analysis (gene per environment analysis, GxE) testing PON1 SNPs association with HDL, LDL, Cholesterol, Triglycerides and AIP phenotypes in anthocyanins and polyphenols intake environments using PLINK v1.07 [29]. The two environmental groups (high and low intake) were defined using the two extreme tertiles of anthocyanins and total polyphenol intake distributions in order to maximize the differences between the two groups. Quantitative traits were analysed as residuals, adjusted for sex, age, BMI and the first 10 principal components calculated using R software [31–35]. For each trait, individuals with missing data were excluded from the analysis. To correct for multiple testing we defined a Bonferroni threshold of p = 0.0028. Furthermore, in order to identify the genotypes that are associated to CVD protection (high HDL, low non-HDL cholesterol, triglycerides and AIP) in high anthocyanins/polyphenols intake, we performed a multivariate regression analysis in high and low environment separately using STATA (Stata corp.2015. *Stata Statistical Software: Release 14*. College Station, TX: StataCorp LP). Analyses were adjusted for BMI, age, gender and principal components.

## Results

**Fig. 1:**
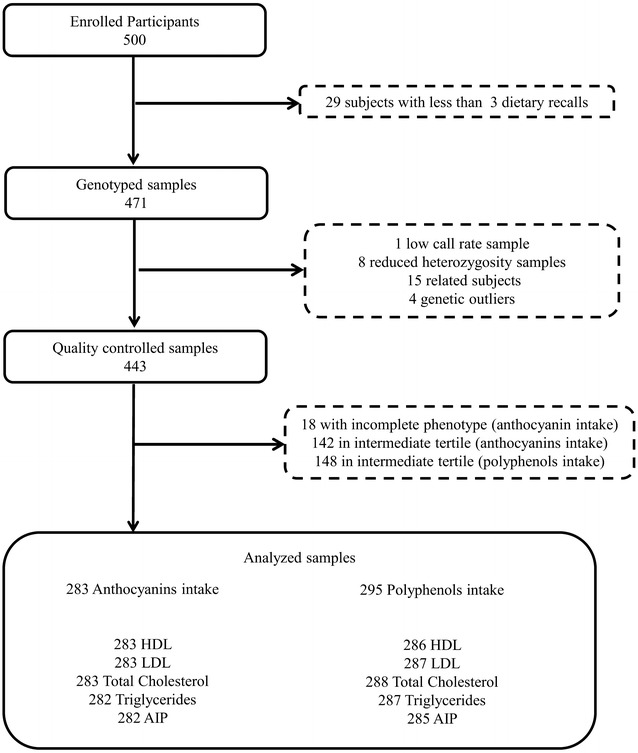
Participant flow chart

Four hundred and forty-three subjects, 175 males and 268 females, were fully eligible for the analysis after quality control of genetic and phenotypic data (Fig. 1).

Among the 500 enrolled participants, 29 subjects with less than three dietary recall interviews were excluded, leaving 471 subjects for genotyping and downstream analysis.

After genotyping, one subject was excluded for low call rate (≤0.95) and eight subjects were filtered out for a reduced proportion of heterozygosity. We then removed 15 related individuals and four genetic outliers after PCA, defined as individuals that exceed six standard deviations from the whole sample along any of the principal components (Additional file 1: Figure S1).

Anthropometric characteristics of the overall sample and of each sub-group identified according to anthocyanins or polyphenols intake are described in Table 2. Anthocyanins intake subgroup included 283 individuals while polyphenols subgroup included 295 individuals. Subjects with missing biochemical data were excluded from each specific analysis (Table 2).

**Table 2:** Characteristics of the study sample according to the different environment subgroups (anthocyanins and polyphenols intake)

Characteristics	Total sample	Anthocyanins intake		Polyphenols intake	
		High	Low	High	Low
Male (%)	40	41	33	37	49*
Age (years)	51.00 ± 13.96 (443)	53.13 ± 13.83 (141)	47.63 ± 13.57 (142)*	49.60 ± 13.60 (148)	49.54 ± 14.38 (147)
BMI	24.70 ± 4.80 (443)	24.62 ± 4.96 (141)	25.21 ± 5.05 (142)	23.86 ± 4.07 (148)	25.79 ± 5.03 (147)*
Obese subjects, BMI ≥30 (%)	12.9	10.6	18.3	11.5	17
HDL (mg/dl)	54.79 ± 13.30 (428)	54.94 ± 12.85 (141)	54.80 ± 13.27 (142)	55.97 ± 14.45 (143)	53.99 ± 13.70 (143)
<35 mg/dl men, <45 mg/dl women (%)	8.6	9.2	12.7	3.5	13.99*
LDL (mg/dl)	125.17 ± 34.51 (429)	128.09 ± 36.77 (141)	124.83 ± 33.27 (142)	121.32 ± 35.26 (143)	126.00 ± 31.90 (144)
≥150 mg/dl (%)	22.1	24.1	21.8	18.8	22.9
Total cholesterol (mg/dl)	201.42 ± 39.28 (430)	203.55 ± 41.54 (141)	200.68 ± 40.64 (142)	197.74 ± 39.46 (143)	203.47 ± 37.98 (145)
>220 mg/dl (%)	30.9	32.4	32.6	29.4	32.4
Triglycerides (mg/dl)	106.32 ± 60.50 (427)	102.56 ± 45.80 (141)	105.69 ± 62.16 (141)	102.64 ± 58.24 (142)	114.62 ± 67.61 (145)
>190 (mg/dl) (%)	8.9	5.7	9.2	9.9	11.1
AIP	−0.11 ± 0.26 (421)	−0.118 ± 0.24 (141)	−0.11 ± 0.26 (141)	−0.14 ± 0.26 (142)	0.08 ± 0.27 (143)

Data are reported as mean ± standard deviation (number of subjects) or percentages. Between-groups comparison of continuous variables was performed using one-way analysis of variance (ANOVA); categorical data were compared between groups using the chi2 test or fisher’ exact test

*AIP* atherogenic index of plasma calculated as [log(triglycerides/HDL)]

* P < 0.05 of the comparison among environment subgroups

Daily anthocyanins intake ranged between 0 and 5.98 mg/day in the lower and 25.7 and 614.4 mg/day in the higher tertile. Polyphenols intake ranged between 99.4 and 804.5 mg/day in the lower and between 1288.0 and 4342.2 mg/day in the higher tertile.

High and low sub-groups were significantly different for age in anthocyanins and for sex (Chi squared test p < 0.05) and BMI in polyphenols environment (t test p < 0.05) (Table 2). BMI did not significantly correlate with anthocyanins intake (r = −0.05, p = 0.3) whereas it weakly correlated with polyphenols intake (r = −0.2, p < 0.0001) (Additional file 2: Figure S2). Interestingly, without considering the genetic background, no significant difference was observed for HDL, LDL, cholesterol, triglycerides and AIP between high and low anthocyanins and polyphenols intake. Evaluating the frequency of subjects out of the physiological ranges for each lipid parameter, we observed that only the percentage of subjects with HDL <45 in females and <35 in males is significantly higher in the low polyphenols intake sub-group compared to the high sub-group (odds ratio 4.4, CI 95 % 1.6–12.1, p = 0.004 for low intake) (Table 2).

Quantitative trait interaction results for the 18 selected SNPs in PON1 and CVD biomarkers, considering anthocyanins and polyphenols intake as environment, are presented in Table 3. According to the number of analyzed SNPs, the Bonferroni threshold is p = 0.0028. A significant p value indicates a difference in association with the lipid profiles between the two environments. The interaction analysis for HDL showed that 2 SNPs had a significant p value in interaction with anthocyanins (rs854549 flanking the gene at the 3′, p = 0.0008 and rs854552, at the 3′ untranslated region (3′ UTR) p = 0.001) and 2 SNPs in interaction with polyphenols (rs854571, p = 0.0021 and rs854572 p = 0.0020, both in the promoter region of the gene). A significant interaction model was found for rs854551, located at 3′ UTR (p = 0.0022) in relation to AIP and anthocyanins and for the intronic rs3917477 (p = 0.0026) in relation to total cholesterol and polyphenols.

**Table 3:** Results of the interaction analysis between anthocyanin and polyphenols intake and PON1 SNPs on HDL, LDL, cholesterol, triglycerides and AIP

SNP	Anthocyanins intake					Polyphenols intake				
	HDL	LDL	Cholesterol	Triglycerides	AIP	HDL	LDL	Cholesterol	Triglycerides	AIP
rs854549	*0.0008*	0.3941	0.1031	0.6548	0.1787	0.0998	0.5160	0.2006	0.5240	0.9975
rs3735590	0.3448	0.1405	0.0397	0.0622	0.3134	0.2444	0.0866	0.0303	0.1277	0.2865
rs854551	0.0042	0.6413	0.8251	0.0103	*0.0022*	0.0206	0.0634	0.0575	0.0064	0.0041
rs854552	*0.0010*	0.6538	0.3149	0.1498	0.0177	0.0071	0.4599	0.6442	0.1186	0.0493
rs3917567	0.2844	0.3614	0.1339	0.1405	0.5745	0.2103	0.2276	0.0400	0.1382	0.5090
rs854555	0.0412	0.7870	0.5833	0.2299	0.1951	0.4876	0.6883	0.7198	0.7992	0.8506
rs3917550	0.0396	0.7860	0.8563	0.4393	0.2520	0.3543	0.5997	0.3524	0.0470	0.1137
rs662	0.3499	0.4358	0.3243	0.9524	0.8667	0.6203	0.5619	0.6976	0.7735	0.5416
rs3917538	0.4642	0.9174	0.9004	0.4727	0.7861	0.2624	0.8660	0.5450	0.5523	0.7498
rs2074354	0.2774	0.9755	0.6970	0.9028	0.3660	0.0362	0.9619	0.8127	0.1865	0.0592
rs854560	0.0083	0.6339	0.3162	0.4117	0.1807	0.1480	0.9995	0.6245	0.9061	0.5260
rs3917498	0.2024	0.8515	0.7380	0.3965	0.4059	0.7842	0.9222	0.9400	0.9443	0.9068
rs2074351	0.2300	0.5031	0.5218	0.1089	0.1844	0.4547	0.6098	0.2953	0.4237	0.8078
rs2272365	0.6519	0.4859	0.2930	0.2423	0.6737	0.1485	0.5808	0.4599	0.4784	0.3204
rs854569	0.3324	0.4350	0.3155	0.9161	0.7891	0.0071	0.6101	0.0961	0.5367	0.6539
rs3917477	0.9098	0.0364	0.0437	0.4110	0.6409	0.0701	0.0674	*0.0026*	0.0544	0.4517
rs854571	0.1042	0.3670	0.1272	0.3527	0.9355	*0.0021*	0.1417	0.0067	0.4772	0.4889
rs854572	0.0087	0.8026	0.2546	0.6123	0.3915	*0.0020*	0.5219	0.1108	0.8518	0.2163

Covariates included age, sex, BMI and the first 10 principal components. P values reaching the Bonferroni threshold (p = 0.0028) are highlighted in italics

**Fig. 2:**
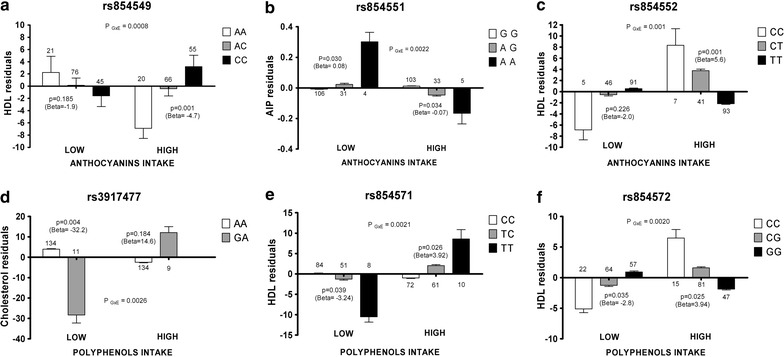
Phenotypes distribution relative to genotypes at best SNPs according to anthocyanins and polyphenols intakes. rs854549 (**a**), rs854551 (**b**), rs854552 (**c**), rs3917477 (**d**), rs854571 (**e**) and rs854572 (**f**).* Y axis* reports the residuals calculated for each phenotype (HDL, AIP and cholesterol) adjusted for age, sex, BMI and the first ten principal components. The *bars* summarize the distribution as mean and standard errors. For each* bar*, the numbers of individuals per *genotype* are indicated. P_GxE_ denotes the SNP x environment interaction analysis comparing high and low intake both for anthocyanins (**a**–**c**) and polyphenols (**d**–**f**). *P* indicates the multivariate linear regression analysis comparing genotypes in each environment subgroup (*low*/*high*); Beta coefficients refer to minor alleles

Among the significant GxE interactions with HDL, cholesterol and AIP, we then selected the genotypes associated to CVD protection in the high anthocyanins and polyphenols intake. Figure 2 shows the trend of lipid profiles across the three genotypes for each significant GxE SNP in low or high environment. In particular, in high anthocyanins intake, HDL concentration was higher in carriers of the major CC genotype for rs854549 (p = 0.001, Beta = 4.7) and in the minor CC genotype for rs854552 (p = 0.001, Beta = 5.6). AIP was lower in minor AA carriers for rs854551 (p = 0.034, Beta = −0.07). Considering high polyphenols intake, HDL concentration was higher in minor TT carriers for rs854571 (p = 0.026, Beta = 3.92) and higher in minor CC compared to GG for rs854572 (p = 0.025, Beta = 3.94). Rs3917477 was not significantly associated to cholesterol in high polyphenols intake.

## Discussion

Within the ATHENA project, we performed a nutrigenetic observational study to determine whether SNPs that describe the genetic variability in PON1 gene can influence the response of cardiovascular health biomarkers to polyphenols and anthocyanins. We collected genetic, dietary, environmental, lifestyle data and laboratory measurements in 443 healthy Italians. As protective biomarkers of cardiovascular health we considered high HDL, low total cholesterol, LDL, triglycerides and AIP [14, 25].

In our analysis, high and low antioxidant intakes did not exert any beneficial effect on the target phenotypes if the genetic background related to PON1 gene was not considered. These results are in line with previous interventional studies that showed discordant findings on the effect of anthocyanins on common biomarkers of CVD [14]. On the contrary, using a nutrigenetic approach, we could identify 5 SNPs significant at Bonferroni level (rs854549, rs854551, rs854552, rs854571, rs854572) and for each SNP we pointed out the genotype with a significant cardiovascular protective effect under high antioxidants intake.

In high anthocyanins intake, carriers of the C protective allele at rs854549 experienced an increase in HDL levels of 4.7 mg/dl (p = 0.001) while carriers of the C protective allele at rs854552 showed an increase of 5.6 mg/dl (p value 0.001). Considering high polyphenols intake, HDL levels were 3.92 mg/dl higher in T carriers for rs854571 (p = 0.026) and 3.94 mg/dl higher in C carriers for rs854572 (p = 0.025). These effects are of note if we consider that Boes [36] estimated that an increase of 1 mg/dl of HDL levels is associated with a 2 and 3 % reduction of the risk for coronary artery disease in men and women, respectively. AIP was lower in A carriers for rs854551, with a decrease of 0.07 (p = 0.034) in high anthocyanins intake.

PON1 gene is associated with several human diseases, related to oxidative stress including cardiovascular disease, Parkinson’s disease and cancer [6] and is inversely associated to the risk of CVD, particularly to atherosclerosis [37].

PON1 enzyme is tightly associated with HDL particles and protects both LDL and HDL from oxidation, a major step in the progression of atherosclerosis, the underlying pathophysiologic factor for the majority of cardiovascular diseases [37–39]. HDL contributes to PON1 enzyme stabilization, furnishes a hydrophobic environment that could be important for PON1 function and is a key player in the reverse cholesterol transport, which shuttles cholesterol from peripheral cells (e.g. macrophages) to the liver or other tissues.

As lifestyle determinants such as smoking, alcohol intake and exogenous or endogenous oxidants can modify PON1 levels and activity, several strategies were used to test if antioxidant supplementation, including polyphenols and anthocyanins, could improve PON1 function. It has been demonstrated that anthocyanins and polyphenols promote antioxidant activity and cholesterol efflux capacity of HDL. They also enhance PON1 stabilization, its association with HDL and catalytic activity [15, 16, 40].

Rs854549, that we found associated to HDL in interaction with anthocyanins intake, is a 3′ flanking variant, repeatedly reported as tagger SNP for PON1 and as modulator of PON1 activities [41, 42].

Huen et al. reported rs854551 and rs854552, both mapping at the 3′ UTR of the gene, as significantly associated to paraoxonase activity of PON1 in a study on Mexican Newborn and Mothers [42].

Rs854571 and rs854572 map in the promoter region of PON1 gene and have been independently reported to produce an approximatively two-fold change in PON1 expression levels in human hepatoma cell line HepG2 [38, 43]. Leviev [43] reported a significant increase in activity of PON1 promoter related to the T allele of rs854571, the same allele that we found associated to higher level of HDL in high anthocyanins intake. Brophy [38] showed a significant increase in activity of PON1 promoter related to the G allele of rs854572, that in our sample was associated to lower HDL levels in high anthocyanins intake. A recent genome-wide association analysis demonstrated that PON1 polymorphisms are strongly associated to PON1 function, and especially rs854572 is the best predictor of arylesterase activity [20].

On the contrary, associations between PON1 SNPs and lipid profiles have been mostly controversial [21–23, 36, 44–46]. Nus et al. [23] evaluated the effect of walnut enriched meat consumption on lipid profile, in subjects at increased risk of CVD, according to rs662 and rs854560 genotypes. They observed that rs662 TT carriers had lower HDL, LDL and triglycerides levels compared to C carriers and that the difference increased if the SNPs were considered in interaction (rs662 TT + rs854560 TT + AT versus the other genotypes). However, the lipid profile did not differ between rs662 and rs854560 after consumption of either walnut enriched or control meat diet. De Souza et al. in a pharmacogenomics study on statin response in Brazilians, found that T allele carriers of rs854560 reached their HDL lipid target (HDL >1.55 mmol/L) more often than patients who were homozygous AA. Rios et al. [44]. showed rs662 as associated to triglycerides and HDL levels in male Caucasian-Brazilians. Van Himbergen et al. [45] found that genetic variants in PON1 associated with high levels and activity of the enzyme, were also associated with higher HDL levels. That study, however, was performed in patients with familial hypercholesterolemia with a high prevalence of atherosclerosis in the population. Van Aalst-Cohen et al. [46] in a familial hypercholesterolemia study and Blatter Garin et al. [21] in a case control study on coronary artery disease could not find any association between the two coding polymorphisms rs854560 and rs662 and HDL level. In our study, rs854560 was significantly associated to HDL in interaction with anthocyanins (p = 0.0083) but the significance was not confirmed after correction for multiple testing. There was a tendency for TT homozygous to higher HDL levels in high anthocyanins intake. Rs662 was not associated to any of the phenotype tested.

Though a limitation of our study could be the small sample size, its strength is an accurately phenotyped cohort that includes subjects with well-established long-term dietary habits regarding antioxidant intake. To address bias due to self-reported data from the nutritional interview, trained dieticians conducted the 24 h dietary recall using pictures (i.e. validated Food Atlas) to help people identifying food portions. In addition, as seasonality affects anthocyanins levels that are higher in summer and lower in winter, we repeated the 24 h recall interview at least three times a year and used the average yearly intake for all the analyses [47, 48]. This has increased the precision in evaluating the chronic effect of the diet on lipid profile. Moreover, the DMS software, integrating different databases, returns a detailed estimate of the anthocyanins and polyphenols content for all the recorded foods. A validation on an independent sample would allow confirming causality of the SNPs identified in the present study.

## Conclusions

This nutrigenetic study highlights the role of PON1 as susceptibility gene for cardiovascular health under high antioxidant intake. We identified protective genotypes in 4 independent polymorphisms, presenting a significant association with increased HDL level under high polyphenols and anthocyanins intake. The identified alleles could be used to stratify subjects who can benefit from high antioxidant consumption to improve their HDL levels.

## Additional files

10.1186/s12967-016-0941-6 Principal component plot of discovery sample.
10.1186/s12967-016-0941-6 Correlation between (a.) BMI and anthocyanins intake and (b.) BMI and polyphenols intake. “r” refers to Spearman’ correlation coefficient.

## Abbreviations

AIP: atherogenic index of plasma
ATHENA: anthocyanin and polyphenols bioactive for health enhancement through nutritional advancement
BDA-IEO: Banca Dati Alimentare-Istituto Europeo Oncologico
CVD: cardiovascular disease
DMS: diet monitoring solution
GxE: gene per environment analysis
HepG2: human hepatoma cell line
IBD: identity by descent
MAF: minor allele frequency
PCA: principal component analysis
PON1: paraoxonase 1
SNP: single nucleotide polymorphism
UTR: Untranslated Region
