# Supplementary figures and images for: Interaction between polyphenols intake and PON1 gene variants on markers of cardiovascular disease: a nutrigenetic observational study
Source: J Transl Med. 2016 Jun 23;14:186. doi: 10.1186/s12967-016-0941-6 (PMC4918189; doi:10.1186/s12967-016-0941-6)

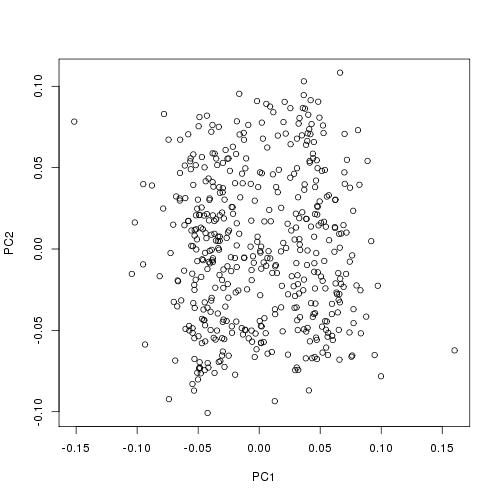

Supplement: Supplementary file 1 — 10.1186/s12967-016-0941-6 Principal component plot of discovery sample. [file 12967_2016_941_MOESM1_ESM.jpg]

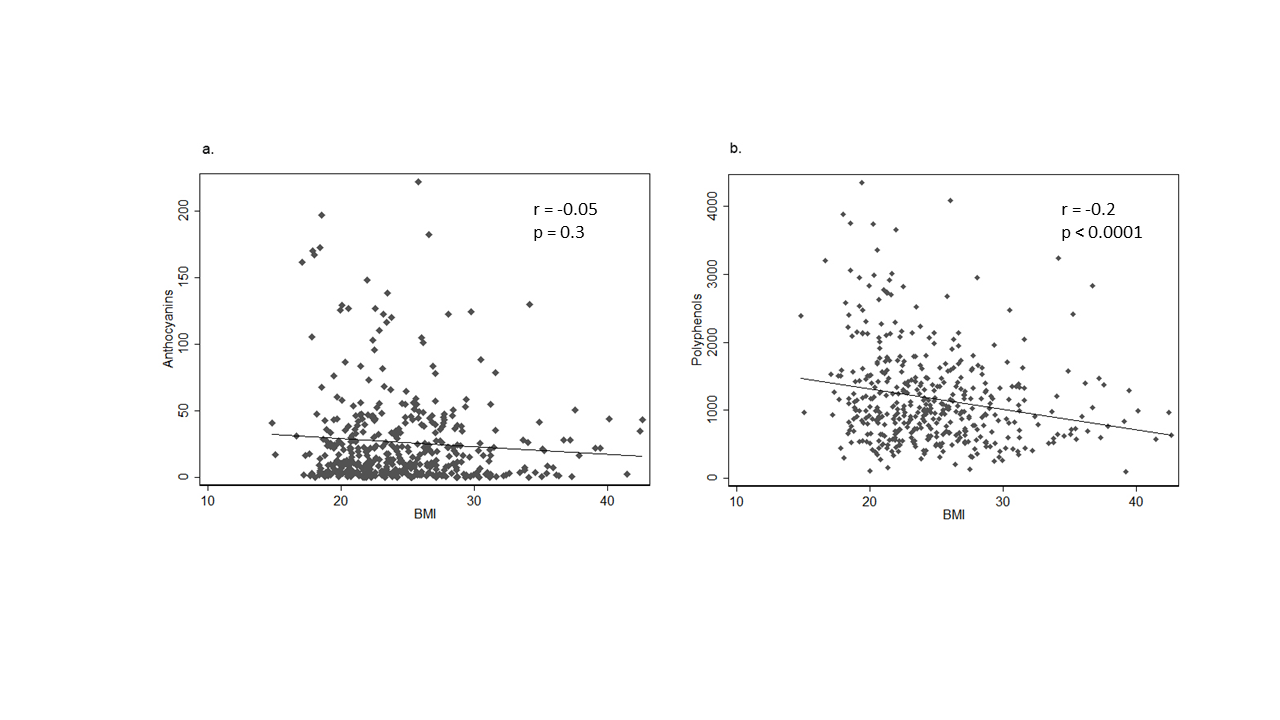

Supplement: Supplementary file 2 — 10.1186/s12967-016-0941-6 Correlation between (a.) BMI and anthocyanins intake and (b.) BMI and polyphenols intake. “r” refers to Spearman’ correlation coefficient. [file 12967_2016_941_MOESM2_ESM.tif]
